# Clustering Sparse Data With Feature Correlation With Application to Discover Subtypes in Cancer

**DOI:** 10.1109/access.2020.2982569

**Published:** 2020-03-26

**Authors:** JIPENG QIANG, WEI DING, MARIEKE KUIJJER, JOHN QUACKENBUSH, PING CHEN

**Affiliations:** 1 Department of Computer Science, Yangzhou University, Yangzhou 225127, China; 2 Department of Computer Science, University of Massachusetts Boston, Boston, MA 02125, USA; 3 Centre for Molecular Medicine Norway, University of Oslo Faculty of Medicine, 0318 Oslo, Norway; 4 Department of Biostatistics, Harvard T. H. Chan School of Public Health, Boston, MA 02115, USA

**Keywords:** Cancer subtype, feature interaction network, similarity metric, somatic mutational data

## Abstract

In this paper, given data with high-dimensional features, we study this problem of how to calculate the similarity between two samples by considering feature interaction network, where a feature interaction network represents the relationship between features. This is different from some traditional methods, those of which learn similarities based on a sample network that represents the relationship between samples. Therefore, we propose a novel network-based similarity metric for computing the similarity between samples, which incorporates the knowledge of feature interaction network, in order to overcome the data sparseness problem. Our similarity metric uses a new Feature Alignment Similarity measure, which does not directly compute the similarities among samples, but projects each sample into a feature interaction network and measures the similarities between two samples using the similarities between the vertices of the samples in the network. As such, when two samples do not share any common features, they are likely to have higher similarity values when their features share the similar network regions. For ensuring that the metric is useful in a real-world application, we apply our metric to discover subtypes in tumor mutational data by incorporating the information of the gene interaction network. Our experimental results from using synthetic data and real-world tumor mutational data show that our approach outperforms the top competitors in cancer subtype discovery. Furthermore, our approach can identify cancer subtypes that cannot be detected by other clustering algorithms in real cancer data.

## I. INTRODUCTION

Clustering is a key task in data mining, in which data samples are grouped into clusters. Sample within one cluster are more similar than those in different clusters. Many clustering algorithms are good at handling low dimensional data, involving only several dimensions. It is challenging to cluster data samples in a high dimensional space, especially considering that such data can be very sparse and highly skewed [1]–[3]. High-dimensional data is a phenomenon in real-world data mining applications. Gene data is a typical example. The total number of unique genes in a gene data set represents the number of dimensions, which is usually in the thousands. High-dimensional data occurs in text data and business data as well [4]. Sparsity is an accompanying phenomenon of high-dimensional data.

Clearly, clustering of high-dimensional sparse data requires special treatment. There are three strategies to alleviate problems caused by the sparsity and high dimensionality of the data. The first one computes semantic similarity between samples by using an external knowledge source, such as WordNet [5] and Word2Vec [6] in documents. However, these methods are domain dependent and language dependent. The second one used methods which are co-clustering based, in which the features and the samples are simultaneously clustered by exploiting the duality between them [7], [8]. However, the methods rely solely on the feature distributions to cluster the samples and vice-versa. The last one uses a sample network that represents the relationship between samples to improve similarity estimates, e.g. social network [9], [10], citation network [11]. These methods are influenced by the structure of the sample network.

**FIGURE 1.**
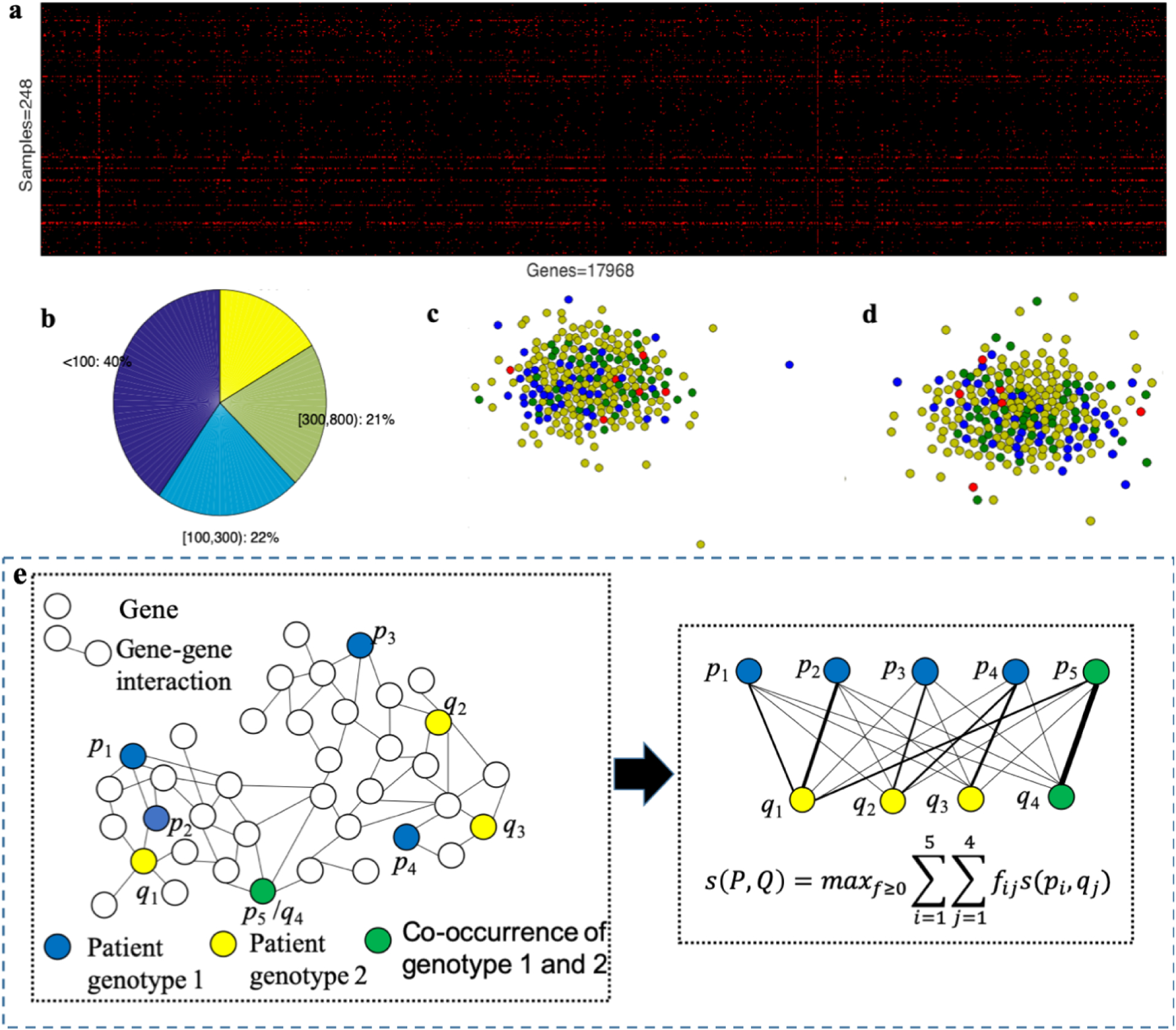
Overview of the challenges of discovering subtypes in uterine cancer data. (a) The heat map of uterine cancer data (248 × 17.968). The red dot means the gene is mutated in the sample. (b) The statistics of samples. (c) Visualization of uterine cancer data using T-SNE. Uterine cancer data can be divided into four recorded subtypes on a histological basis. Different colors represent different subtypes. (d) Visualization of the 50-dimensional results of NMF method using T-SNE. (e) Example illustrating two patient somatic mutation profiles (P and Q) over a molecular interaction network. Mutated genes are shown in blue (patient 1) and yellow (patient 2) in the context of a gene interaction network. Based on the gene network, genes with high scores in both patients are connected by a bold line. The similarity of two patients is calculated by aligning the weight of genes of *P* to genes of *Q* that attempts to maximize the objective function shown, where *s*(*p*_*i*_, *q*_*j*_) represents the similarity of two genes and *f*(*i*, *j*) represents how much the weight of *p*_*i*_ matches *q*_*j*_.

In contrast to the above strategies, the novel similarity metric we are studying computes the similarity between samples by incorporating the knowledge of a feature interaction network, where feature interaction network consists of the features of the samples in data. In some applications, there exists such a feature interaction network. For example, in the task of subtype discovery from gene mutational data, a gene interaction network contains the relationship between genes which can help to alleviate the problem of sparseness [12]–[14]. Here, we give an overview of the challenges of discovering subtypes from real uterine cancer mutation data, which are listed in Figure 1.

1. Few samples and high dimensional features. Figure 1(a) shows the heat map of uterine cancer data, which includes 248 patients over 17,968 unique genes. As collecting such data is usually prohibitively expensive, only hundreds of samples for each cancer can be obtained. The data is high dimensional with 17,968 features.
2. Sparse and heterogeneous characteristics. From Figure 1(a), we can see that some rows (samples) include many mutated genes and some rows only have a few mutated genes. Figure 1(b) gives the statistics of the number of samples with the number of mutated genes in each sample. The total number of genes is 17,968 and the percentages of samples that contain less than 100 are 40%, which means the data is very sparse. Compared with the 40% (<100), the percentages of samples that contain greater than 800 are 21%, which indicates the data is heterogeneous.
3. Discovering the process using the mutation data for subtype discovery is a challenging task. A good patient representation is expected to group similar patients and separate the different groups. Figure 1(c) shows the T-SNE based visualization [15] of the uterine cancer data we have. In medicine, Uterine data is usually divided into four recorded subtypes based on a histological basis. Four different colors represent four different subtypes in Figure 1(c). However, we cannot distinguish the four different groups using the original data representation. Figure 1(d) shows patient representations using Non-negative Matrix Factorization (NMF) [12], [16], and we can draw a similar conclusion of distinguishing the four different groups because the graph in Figure 1(d) resembles the graph in Figure 1(c). Thus, we can conclude NMF is unsuited to gene mutation data, even though NMF can successfully discover subtypes from gene expression data.

Therefore, in this paper, we try to overcome the problems of traditional similarity-based metrics when applied to high-dimensional sparse data, e.g., gene mutational data. We propose a new network-based similarity metric to take advantage of the prior knowledge of a feature interaction network. We project each sample into the feature interaction network and measure the similarity between two samples using the similarity between features in the network in Figure 1(e). In contrast to traditional similarity metric, our metric generates a high similarity value of two samples when their features share similar network regions, even if they do not share any common features. Experimental results show that our approach outperforms the top competitors in cancer subtype discovery using a comprehensive set of evaluation metrics. Furthermore, our approach can identify cancer subtypes with biological significance that cannot be detected by other clustering algorithms using real cancer data.

Thus, our main contributions are as follows:

1. Network-based similarity metric: we propose a novel similarity metric to measure the similarity between samples using a feature interaction network.
2. Effectiveness: When applying subtype discovery, our approach outperforms state-of-the-art algorithms in discovering cancer subtypes, and detects biologically significant cancer subtypes that cannot be identified by other top competitors using real cancer data.

Furthermore, our network-based similarity metric can be easily incorporated into any clustering algorithm that contains data attributes with network structures.

The paper is organized as follows: Section 2 discusses related work; Section 3 presents the proposed metric for computing the similarity between samples; Section 4 reports experimental results on synthetic data and uterine adenocarcinoma datasets; Section 5 concludes the paper.

## II. RELATED WORK

We discuss existing work on similarity-based metrics, network-based similarity metrics, and subtype discovery in cancer.

### A. SIMILARITY-BASED METRIC

There are some traditional similarity functions such as Euclidean distance, Cosine similarity and Pearson’s distance, which provide a way to measure how close two samples are [6]. In probabilistic models, data elements can belong to more than one topic, and associated with each element is a set of membership levels, e.g. Non-negative Matrix Factorization (NMF) [12], [16] and Latent Dirichlet allocation (LDA) [17], [18]. Recent approaches for learning sample representations are distributed representations which encode a sample as a compact, dense and lower-dimensional vector with the semantic meaning of a sample distributed along dimensions of the vector. Many neural network-based distributed representation models have been proposed [19], [20] and shown to be able to learn better representations in image datasets and document datasets. The above methods are not very well suited for dealing with high-dimensional sparse data due to sparsity. Besides computing the similarity between samples, the features and the samples in co-clustering are simultaneously clustered by exploiting the duality between them [7]. However, the method relies solely on the feature distributions to cluster the samples and vice-versa.

### B. NETWORK-BASED SIMILARITY METRIC

In practice, there are many datasets that contain explicit relations among samples, such as citation network datasets [11] and NELL dataset [21]. The relationship between samples can be represented as a network. Through utilizing both data and networks, many similarity metrics were proposed [22], [23] and named as network-based similarity metrics, which have been successfully applied in many application domains. In recent years, many models have been proposed to learn lower-dimensional vectors from network and data [9], [10]. But, these methods are unsuited to our problem of incorporating knowledge from a feature interaction network because the network adopted by the above methods is the relationship between samples, whereas the feature interaction network represents the relationship between features.

### C. SUBTYPE DISCOVERY IN CANCER

Identifying cancer subtypes is essential for a wide range of applications, that of which includes a better understanding of the biological complexity of the disease and developing targeted, precision medicine therapeutic interventions [24], [25]. Clustering algorithms are often used for cancer subtype discovery. Subtype discovery is a fundamental yet unsolved problem in cancer analysis as the presence of multiple subtypes can confound many analyses [26].

Gene mutational data, which can be more reliably obtained than gene expression data, help to determine how the subtypes develop, evolve and respond to therapies [27]–[29]. In contrast to dense continuous-value gene expression data, which most existing cancer subtype discovery algorithms use, somatic mutational data are extremely sparse and heterogeneous. This is because there are less than 0.5% mutated genes out of 20,000 human protein-coding genes. Additionally, identical mutated genes are rarely shared by cancer patients [13]. The major barriers for clustering algorithms are efficient utilization of extremely sparse and high dimensional gene mutational data in discrete 1 and 0 values.

If we focus on clustering algorithms to stratify sparse and heterogeneous somatic mutational profiles, perhaps the most popular approach for subtype discovery is NMF, which does not require any prior knowledge of the expected number of subtypes or the associated mutational patterns [12]. NMF aims to find two non-negative matrices whose product provides a good approximation to the original matrix. One of its drawbacks is that it does not always result in meaningful parts-based clustering representations. Several researchers addressed this problem by incorporating sparseness constraints (sparse NMF) on one or both non-negative matrices [30], [31]. Likewise, we used NetNMF, which is a NMF variant, to encode the geometrical structure in the data and subsequently regularize one of the two non-negative matrices [32]. NMF has been applied to recover meaningful biological information from cancer-related microarray data without supervision [12], [33]. Even when using sparseness constraints, however, NMF cannot effectively stratify somatic mutation data because of its extremely sparseness. Network-based stratification (NBS) [13] was developed to adopt NetNMF because of the variety of gene interaction networks. So far, NBS is the most effective method to stratify patients in an unsupervised fashion from somatic mutation data. However, its performance still needs significant improvement for a practical clinical application.

## III. PROBLEM DEFINITION

We assume that the data *X* to be analyzed consists of high dimensional binary features and there exists a feature interaction network *G* that represents the relevance between features. Excluding sparse and heterogeneous characteristics, we focus on this type of dataset with the non-overlapping characteristic.

*Definition 1 (Non-Overlapping Characteristic):* Two samples who belong to the same topic may not share identical mutated genes.

*Defintion 2 (Sparse and Heterogeneous characteristics):* The dataset can be represented as a binary matrix of feature attributes $X\in B^{M\times N}$, where *M* is the number of sample data and *N* is the number of features. The sparse characteristic describes the scenario where most of the sample data contains few non-zero features. Additionally, the number of non-zero features is far less than the totoal number of features *N*. The heterogeneous characteristic describes the scenario where some of the samples contains hundreds of non-zero features, especially in comparison to the sparse characteristic.

*Defintion 3 (Feature Interaction Network):* The feature interaction network *G* = (*V*, *E*) consists of *N* features as nodes, where *V* = {*v*_1_, …, *v*_*N*_} represents the set of vertices, *E* represents the set of edges, and each vertex represents one feature. Each edge connecting two vertices *v*_*i*_ and *v*_*j*_ has a weight which represents the relevance between two vertices, denoted as *s*(*v*_*i*_, *v*_*j*_). So if the weight of the edge that connects two vertices is high, then the two vertices are more relevant. Here, *v*_*i*_ and *v*_*j*_ are referred as neighbors. *Edges*(*v*_*i*_) represents all edges connecting to vertex *v*_*i*_ and *Neigh*(*v*_*i*_) represents all neighbors of vertex *v*_*i*_.

Based on these characteristics, some common metrics are not fit for this type of data. Therefore, considering the knowledge existing in feature interaction network *G*, we introduce Feature Alignment Similarity (FAS) which is a new network-based similarity metric that embeds intrinsic relevance among features. FAS is designed to deal with data samples that have few overlapping features. Datasets with non-overlapping features are common in text data and biological data. For example, two cancer patients who belong to the same cancer subtype may not share identical mutated genes due to the complicated nature of cancer diseases [13]. In natural language processing, it is common that many short texts (e.g., Tweets or Comments) which use uncommon words can still discuss the same topic [31]. However, those non-overlapping features still exhibit a level of similarity because those features are related to each other through a feature interaction network. We now formally define the relevant concepts.

If we cannot obtain the edge weight beforehand, the relevance between features can be estimated using the network structure explained in Section 3.2. The edge weight only provides the relevance of two neighbors. For two non-neighboring vertices, two vertices that are close in the network can have higher relevance than those that are distant in the network, which is also considered by our method that is explained in Section 3.2.

We formulate the problem of computing the similarity between two data samples as follows.

- Given: two samples *P* and *Q*, and a feature interaction network *G* that describes the network relevance among features.
- Find: a similarity metric that properly integrates the knowledge of feature interaction network.
- Objective: optimal alignment of the features of a sample *P* to the features of the sample *Q* that calculates the maximal similarity between two samples *P* and *Q*.

In the application of cancer subtyping, cancer data is represented as a binary matrix, where 1 means the gene in this patient is mutated. The feature interaction network can be constructed using gene interaction information from gene networks, e.g., PathwayCommons (a resource for biological pathway analysis) [34], STRING (functional protein association networks) [35] and HumanNet (probabilistic functional gene network of Homo sapiens) [36]. A detailed discussion will be provided in Section 4.1.

### A. FEATURE ALIGNMENT SIMILARITY (FAS)

In order to ensure that the maximal similarity between two data samples do not exceed 1, each feature of a data sample has a weight associated with itself, which is defined as 1 over the number of features in this sample, e.g., *w*(*v*_*i*_) = 1/*n*, where *n* is the total number of features, *v*_*i*_ is the *i*th feature. Therefore, features that reside in each data sample are equally important when computing the similarity between two samples.

Consider two data samples $P=\{\left(p_{1},w_{p_{1}}\right),\left(p_{2},w_{p_{2}}\right),\ldots ,\left(p_{m},w_{p_{m}}\right)\}$ and $Q=\left\{\left(q_{1},w_{q_{1}}\right),\left(q_{2},w_{q_{2}}\right),\ldots ,\left(q_{n},w_{q_{n}}\right)\right\}$, where the number of features in *P* is *m*, the number of features in *Q* is *n*, $w_{p_{m}}$ is the weight of *p*_*m*_ in *P*, and $w_{q_{n}}$ is the weight of *q*_*n*_ in *Q*. Here, 1 ≤ *p*_*m*_ ≤ *N* and 1 ≤ *p*_*n*_ ≤ *N*. Our goal is to incorporate the relevance between two features computed by a feature interaction network into the data sample similarity metric. The idea is that two data samples will have a high similarity value when their features share similar network regions in *G*, even if they do not share any common features. First, we allow the weight of each feature *p*_*i*_ in *P* to be transformed into any feature *q*_*j*_ in *Q* in total or in parts. Then, we need to optimally align the weights of the features in the sample *P* to those of sample *Q* to properly calculate the maximal similarity. We define the Feature Alignment Similarity (FAS) as the similarity between samples with non-overlapping features.

*Defintion 4 (Feature Alignment Similarity):* Let $f\in {\mathbb{R}}^{m\times n}$ be an alignment matrix between *P* and *Q*, where *F* = [*f*_*ij*_] represents how much the weight of feature *p*_*i*_ allocates to to feature *q*_*j*_. The similarity of two samples to the maximum cumulative cost required to align all features of one sample to the other sample, namely, $\sum_{i,j}f_{ij}s\left(p_{i},q_{j}\right)$, where *s*(*p*_*i*_, *q*_*j*_) represents the feature relevance that is discussed in subsection 3.2. The maximal similarity between two samples can be calculated using the following objective function,

(1) $$s(P,Q)=\underset{f\geq 0}{\text{max}}\;\sum_{i}^{x}\;\sum_{j}^{y}\;f_{ij}s\left(p_{i},q_{j}\right)\;\text{ where }\sum_{j}^{n}\;f_{ij}=w\left(p_{i}\right)\;\forall i\in \{1,2,\ldots ,m\}\;\sum_{i}^{m}\;f_{ij}=w\left(q_{j}\right)\;\forall j\in \{1,2,\ldots ,n\}$$

which matches all weights of *P* with *Q*, the entire outgoing weight from feature *p*_*i*_ equals *w*(*p*_*i*_), namely $\sum_{j}^{n}f_{ij}=w\left(p_{i}\right)$. Correspondingly, the amount of incoming weight to feature *q*_*j*_ must equal *w*(*q*_*j*_), namely, $\sum_{i}^{m}f_{ij}=w\left(q_{j}\right)\;$. The optimal flow *F* is found by solving this linear optimization problem, and the best average time complexity of solving the FAS problem is O(*N*^3^log*N*), where *N* is the number of all features in the feature interaction network [37]. For a dataset *X* with hundreds of samples, solving the FAS optimization problem for any two sample data in *X* can become prohibitive. Therefore, we will introduce a faster similarity computation method in Section 3.4.

For example, let us consider a special case for two sample data that are the same *P* = {(*a*, 1/3), (*b*, 1/3), (*c*, 1/3)} and *Q* = {(*a*, 1/3), (*b*, 1/3), (*c*, 1/3)} with *s*(*a*, *a*) = 1, *s*(*b*, *b*) = 1, *s*(*c*, *c*) = 1, *s*(*a*, *b*) = *s*(*b*, *a*) = 0.5, *s*(*a*, *c*) = *s*(*c*, *a*) = *s*(*b*, *c*) = *s*(*c*, *b*) = 0, where 1 means two vertices are completely similar and 0.5 means 50% similar. The weight of each feature can be transformed into that of any feature in other samples or many features in other sample. For obtaining the maximal similarity through Equation 1, in this example, the weight of each feature in *P* should be transformed into the corresponding same feature in *Q*. The final similarity of *P* and *Q* is 1/3 × *s*(*a*, *a*) + 1/3 × *s*(*b*, *b*) + 1/3 × *s*(*c*, *c*) = 1.

### B. FEATURE SIMILARITY

In this subsection, we will explain how to compute the similarity between individual features with the help of a feature interaction network *G*, e.g., *s*(*p*_*i*_, *q*_*j*_). For simplification, *s*(*p*_*i*_, *q*_*j*_) is expressed as *s*(*i*, *j*). There are only two cases when calculating the similarity between vertices: the similarity of a vertex with itself and the similarity of two different vertices.

#### 1) SIMILARITY OF A VERTEX WITH ITSELF

In a traditional similarity matrix, the similarity between a feature and itself should be 1. However, in our new similarity metric, the similarity between the same feature from two different samples is calculated based on the different sub-network regions that the feature resides because the same feature from different data samples may exhibit different impacts. We first assign 1 as an initial value to the similarity of one vertex with itself, then we will compute the similarity between other vertices, and then modify the initial value based on their corresponding neighbors in order to distinguish the influence of different vertices, detailed discussion will be provided along with Equations 3, 4, and 5 later in this section.

#### 2) SIMILARITY BETWEEN TWO DIFFERENT VERTICES

The similarity between two vertices is related to the degree of closeness of two vertices. For two different vertices, metric distance between two vertices should become smaller as the similarity increases. The closeness between two vertices is determined by their number of common neighbors. Vertices *i* and *j* are neighbors if they are connected by an edge in the feature interaction network. For two different vertices (*i* and *j*), similarity can be calculated by finding the shortest path from one vertex to another vertex. The shortest path is our proposed measurement of closeness between two features (vertices).

*Defintion 5 (First-Order Proximity):* Edge weight *s*_*ij*_ in *G* are also called first-order proximities between vertex *v*_*i*_ and *v*_*j*_, since they are the first and foremost measures of similarity between two nodes.

At first, for two vertices that are adjacent, its first-order proximity value is set as,

(2) $$s(i,j)=\frac{1}{\left|\text{Edges}(i)\cup \text{Edges}(j)\right|}$$

where *Edges*(*i*) represents all edges connecting to vertex *i*, and | *Edges*(*i*) | represents the number of all edges connecting to vectex *i*.

*Defintion 6 (High-Order Proximity):* The high-order proximity between a pair of vertices describes the proximity of two non-neighboring nodes. The high-order proximity between *v*_*i*_ and *v*_*j*_ is determined by the first-order proximity. Here, the high-order proximity includes second-order proximity, third-order proximity, and so on. The second-order proximity *s*(*i*, *j*) means that vertex *i* and vertex *i* are using one vertex as intermediate point. Correspondently, the third-order proximity means that two vertex are using two vertices as intermediate points.

We will compute the high-order proximity of two vertices by finding the path of greatest similarity using other vertices as intermediate points along the way. We define a function *greatest*(*i*, *j*, *k*) that returns the path of greatest similarity from vertex *i* to vertex *j* using vertices only from the set {1, 2, …, *k*} as intermediate points.

After defining this function, our aim is to find the path of greatest similarity *greatest*(*i*, *j*, *N*) from each *i* to each *j* using only vertices in {1, 2, …, *N*}. In this case, the greatest similar path *greatest*(*i*, *j*, *N*) can represent the similarity between *i* and *j*, namely, *greatest*(*i*, *j*, *N*) = *s*(*i*, *j*).

The path of greatest similarity of each pair of vertices *greatest*(*i*, *j*, *k* + 1) could be either: (1) a path that only uses vertices in the set {1, 2, …, *k*}, or (2) a path that goes from *i* to *k*+1 and then from *k*+1 to *j*.

We know that the best path from *i* to *j* that only uses vertices from 1 through *k* is defined by *greatest*(*i*, *j*, *k*), and it is clear that if there were a better path from *i* to *k* + 1 to *j*, then the length of this path would be the concatenation of the shortest path from *i* to *k* + 1 (only using intermediate vertices in {1, …, *k*} and the path of greatest similarity from *k* to *j* (only using intermediate vertices in {1, …, *k*}.

According to the weight of the edge between vertex *i* and vertex *j*, the base case is,

(3) greatest(i,j,0)=s(i,j)

where base case is the first-order proximity. Consequently, we can define *sim*(*i*, *j*, *k* + 1) recursively,

(4) $$\begin{array}{l} \text{greatest }(i,j,k+1)=\text{max}\;(\text{greatest }(i,j,k),\\ \text{greatest }(i,k+1,k)\;\times \;\text{greatest }(k+1,j,k)) \end{array}$$

Equation 4 ensures that similarity between *i* and *j* is always the path of greatest similarity. This idea is inspired by Floyd’s algorithm which can be used for finding the lowest cost paths in a weighted network [38], [39]. The strategy computes *greatest*(*i*, *j*, *k*) for all (*i*, *j*) pairs for *k* = 1, then *k* = 2, until *k* = *N*, and we can find the path of greatest similarity for all (*i*, *j*) pairs using any intermediate vertices. Finally, the similarity between a vertex *i* and itself can be updated as the sum of its similarity to all of its neighbors vertices in the gene network,

(5) $$s(i,i)=\sum_{j\in \text{neigh}(i)}\;s(j,i)$$

where neigh(*i*) is a neighboring vertex of vertex *i*. The underlying principle of Equation 5 is that similarity between a feature and itself in a densely connected network is greater than in a loosely connected network.

The pseudocode of computing the similarities between features is shown in Algorithm 1. For example, given the five vertices in Figure 1(e), the executed process of the algorithm is shown in Figure 3. Prior to the first iteration of the outer loop, labeled *k* = 0k above, the only known paths correspond to the single edges in the graph. At *k* = 1, paths that go through vertex 1 are found. At *k* = 2, paths going through vertices 1 and 2 are found, and so on. Given two vertices 1 and 4, the path of greatest similarity is [1, 2, 4] before *k* = 5, and the similarity of 1 and 4 is 1/72. When *k* = 5, the path of greatest similarity for 1 and 4 is [1, 5, 4] and the similarity is changed into 1/12.


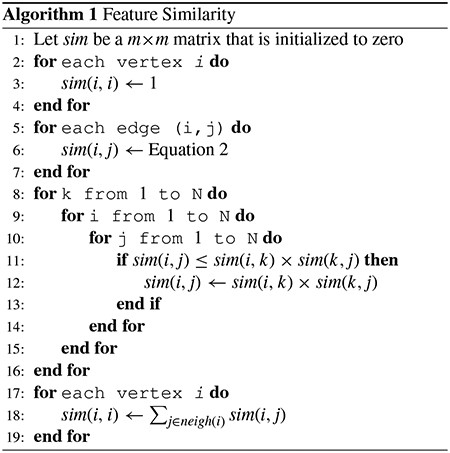


### C. METRIC PROOF

We first prove that the relevance between two features *s*(*i*, *j*) is metric, and then prove that the similarity between two samples *s*(*P*, *Q*) is metric, namely, Feature Alignment Similarity(FAS) is a true metric.

At first, we give the definition of similarity metric.

*Defintion 7 (Similarity Metric* [40]): Given a Set *Y*, a real-valued function s(i, j) is a similarity metric for any *i*, *j*, *k* ∈ *Y*, it satisfies the following conditions:

1. *s*(*i*, *j*) = *s*(*j*, *i*),
2. *s*(*i*, *i*) ≥ 0,
3. *s*(*i*, *i*) ≥ *s*(*i*, *k*),
4. *s*(*i*, *j*) + *s*(*j*, *k*) ≤ *s*(*i*, *k*) + *s*(*j*, *j*)

Condition 1 states that *s*(*i*, *j*) is symmetric. Condition 2 states that for any *i* the self-similarity is nonnegative. Condition 3 states that for any *i* the self-similarity is no less than the similarity between *i* and any *k*, and essentially it means that *i* is always more similar to itself than anything else. Condition 4 states that the similarity between *i* and *j* through *k* is no greater than the direct similarity between *i* and *k* plus the self-similarity of *j*.

*Theorem 8:* Feature Similarity *s*(*i*, *j*) defined in Algorithm 1 is a similarity metric.

Suppose there are three features *i*, *j*, *k* ∈ *V*.

*Proof:* Condition 1, 2 and 3: Symmetry (Condition 1) and non-negativity (Condition 2) hold trivially in all cases. According to Equation 5, Condition 3 holds trivially.

Condition 4: We need to prove *s*(*i*, *j*) + *s*(*j*, *k*) ≤ *s*(*i*, *k*) + *s*(*j*, *j*).

According to Equation 5, we can get *s*(*k*, *k*) ≥ *s*(*i*, *k*) and *s*(*k*, *k*) ≥ *s*(*i*, *j*). The proof can be classified into two cases.

*Case 1: s*(*i*, *k*) ≥ *s*(*i*, *j*)) or *s*(*i*, *k*) ≥ *s*(*j*, *k*);

*Case 2: s*(*i*, *k*) < *s*(*i*, *j*) and *s*(*i*, *k*) < *s*(*j*, *k*).

For case 1, from Condition 3 we can get that *s*(*j*, *j*) ≥ *s*(*i*, *j*) and *s*(*j*, *j*) ≥ *s*(*j*, *k*). From that, it is clearly *s*(*i*, *j*) + *s*(*j*, *k*) ≤ *s*(*i*, *k*) + *s*(*j*, *j*) is true.

**FIGURE 2.**
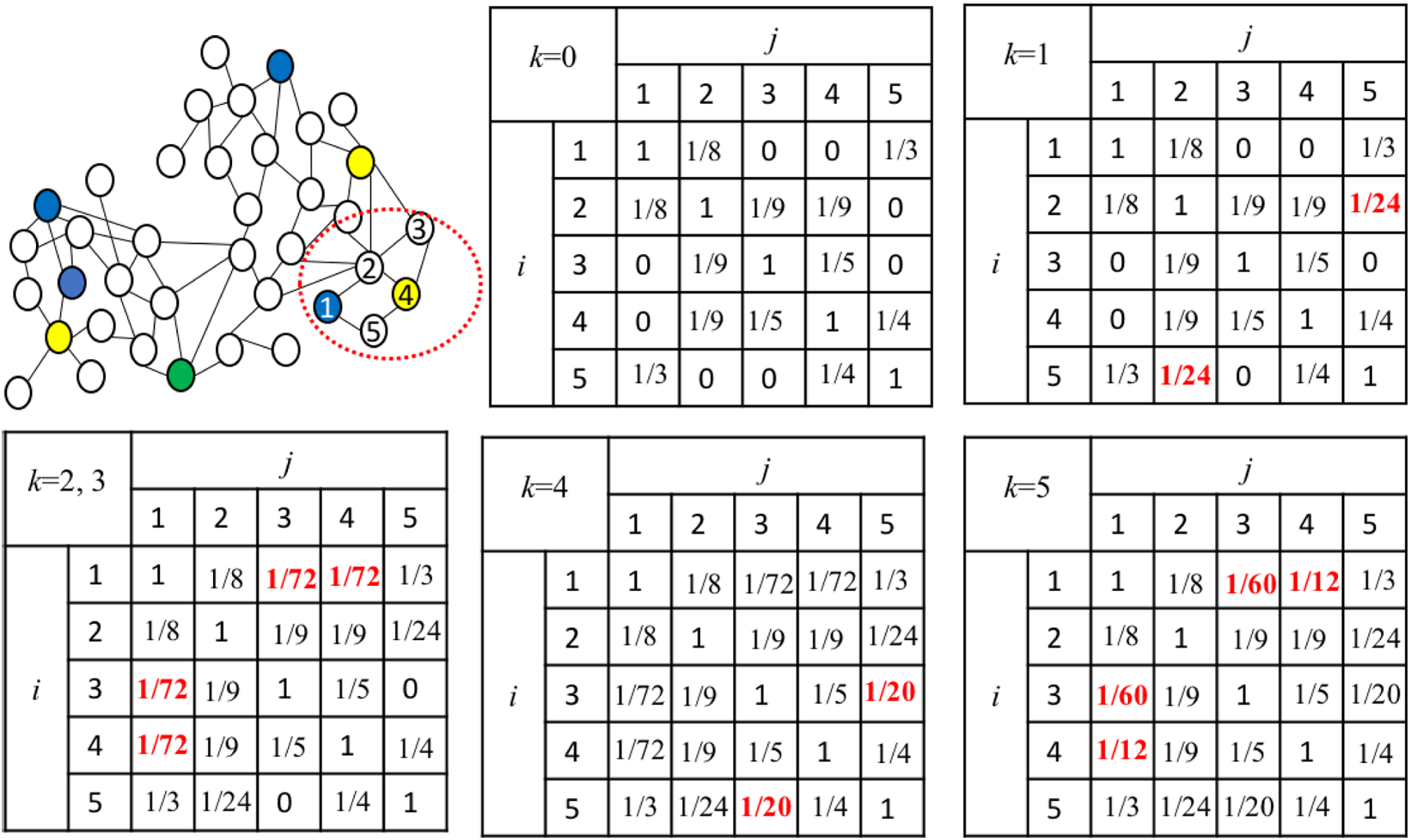
The similarity matrix at each iteration of *k* on part of Figure 1(e), with the updated similarities in red.

For case 2, only when *i* connects *k* through *j*, we have *s*(*i*, *k*) < *s*(*i*, *j*) and *s*(*i*, *k*) < *s*(*j*, *k*). For example, in Figure 2, since 1 connects 4 through 5, we have *sim*(1, 4) < *sim*(1, 5) and *sim*(1, 4) < *sim*(4, 5). Because *j* is the intermediate vertex from *i* and *k*, based on Equation 5, we have *s*(*i*, *k*) + *s*(*j*, *j*) > *s*(*j*, *j*) ≥ *s*(*i*, *j*) + *s*(*j*, *k*).

Therefore, Theorem 8 is true.

*Theorem 9:* Feature Alignment Similarity FAS is a similarity metric.

Given a set of samples *X*, for any (*P*, *Q*, *R*) ∈ *X*, we need to prove FAS satisfies conditions 1–4.

*Proof:* Symmetry (Condition 1) and non-negativity (Condition 2) hold trivially in all cases, so we only need to prove that Condition 3 and Condition 4 hold.

For Condition 3, we only need to prove that *FAS*(*P*, *P*) ≥ *FAS*(*P*, *Q*).

Let *f*_*ij*_ represent how much the weight of feature *p*_*i*_ of *P* flows to feature *q*_*j*_ of *Q*. Based on Definition 4, we can get $\sum_{j}f_{ij}=w\left(p_{i}\right)$.


$$\begin{array}{l} FAS(P,Q)\leq \sum_{i,j}\;f_{ij}s\left(p_{i},q_{j}\right)\leq \sum_{i}\;w\left(p_{i}\right)s\left(p_{i},p_{i}\right)\\ =FAS(P,P) \end{array}$$

For Condition 4, we need to prove that *FAS*(*P*, *Q*) + *FAS*(*Q*, *R*) ≥ *FAS*(*P*, *R*) + *FAS*(*Q*, *Q*).

Except *f*_*ij*_, let *g*_*jk*_ represent how much the weight of feature *q*_*j*_ of *Q* flows to feature *r*_*k*_ of *R*. Now consider the flow from *p*_*i*_ to *q*_*j*_ and then to *r*_*k*_. The largest weight that moves as one unit from *p*_*i*_ to *q*_*j*_ and from *q*_*j*_ to *r*_*k*_ defines a flow which we call *b*_*ijk*_, where *i*, *j* and *k* correspond to *p*_*i*_, *q*_*j*_ and *r*_*k*_ respectively.

From the two constraints $\sum_{j}f_{ij}=w\left(p_{i}\right)$ and $\sum_{j}f_{ij}=w\left(q_{i}\right)$ of Definition 4, the following equations can be obtained:

(6) $$\sum_{k}\;h_{ik}=\sum_{j,k}\;b_{ijk}=\sum_{j}\;f_{ij}=w\left(p_{i}\right),$$

and

(7) $$\sum_{i}\;h_{ik}=\sum_{i,j}\;b_{ijk}=\sum_{j}\;g_{jk}=w\left(r_{k}\right),$$

and

(8) $$\sum_{i}\;f_{ij}=\sum_{i,k}\;b_{ijk}=\sum_{k}\;g_{jk}=w\left(q_{j}\right).$$

Clearly, we have $\sum_{k}b_{ijk}=f_{ij}$ which is the flow from *p*_*i*_ to *q*_*j*_. Likewise, we have $\sum_{i}b_{ijk}=g_{jk}$ and $\sum_{j}b_{ijk}=h_{ik}$. Hence, we have,

(9) FAS(P,R)+FAS(Q,Q)

(10) $$=\sum_{i,k}\;h_{ik}s\left(p_{i},r_{k}\right)+\sum_{j}\;w\left(q_{j}\right)s\left(q_{j},q_{j}\right)$$

(11) $$=\sum_{i,j,k}\;b_{ijk}s\left(p_{i},r_{k}\right)+\sum_{i,j,k}\;b_{ijk}s\left(q_{j},q_{j}\right)$$

(12) $$\geq \sum_{i,j,k}\;b_{ijk}s\left(p_{i},q_{j}\right)+\sum_{i,j,k}\;b_{ijk}s\left(q_{j},r_{k}\right)$$

(13) $$=\sum_{i,j}\;f_{ij}s\left(p_{i},q_{j}\right)+\sum_{j,k}\;g_{jk}s\left(q_{j},r_{k}\right)$$

(14) =FAS(P,Q)+FAS(Q,R)

Equation 9 to 10 and 13 to 14 is based on Definition 4. Equation 11 to 12 utilizes Theorem 8.

Therefore, Theorem 9 is true.

### D. FAST SIMILARITY COMPUTATION

The best average time complexity of solving FAS problem is O(*m*^3^log*m*), where *m* is the number of all nodes in a feature interaction network [37]. With hundreds of samples, solving the FAS optimal problem can become prohibitive. Therefore, we introduce an upper bound of the FAS problem that allows us to prune away the majority of the samples without ever computing the exact FAS similarity. To obtain a much tighter bound, we relax the FAS problem and remove one of the two constraints $\sum_{j}^{n}f_{ij}=w\left(p_{i}\right)\;$ and $\sum_{i}^{m}f_{ij}=w\left(q_{i}\right)\;$ respectively. We are unable to remove both constraints resulting in the trivial upper bound *T* = 1. Here, if we remove the second one, the optimization becomes,

(15) $$s(P,Q)=\underset{f\geq 0}{\text{max}}\;\sum_{i}^{m}\;\sum_{j}^{n}\;f_{ij}s\left(p_{i},q_{j}\right)\;\;\text{such that }:\sum_{j}^{n}f_{ij}=w\left(p_{i}\right)\;\forall i\in \{1,2,\ldots ,x\}$$

This relaxed optimization yields an upper-bound to the FAS similarity, because every FAS solution satisfying both constraints must remain a feasible solution if one constraint is removed. The optimal solution is that each feature in *p*_*i*_ is aligned to the most similar feature in *p*_*j*_. Precisely, an optimal f* matrix is defined as,

(16) $$f_{ij}^{*}=\{\begin{array}{c} w\left(p_{i}\right),\;\;\text{if }j={\text{argmax}}_{j}s\left(p_{i},q_{j}\right)\\ 0,\;\text{ otherwise}. \end{array}$$

Let *f* represent any feasible matrix for the relaxed problem, the contribution to the objective value for any feature *p*_*i*_, with closest gene $q_{j}^{*}={\text{argmax}}_{q_{j}}s\left(p_{i},q_{j}\right)$, cannot be larger:

$$\sum_{j}\;f_{ij}s\left(p_{i},q_{j}\right)\leq \sum_{j}\;f_{ij}s\left(p_{i},q_{j}^{*}\right)=s\left(p_{i},q_{j}^{*}\right)\sum_{j}\;f_{ij}\;=s\left(p_{i},q_{j}^{*}\right)w\left(p_{i}\right)=\sum_{j}\;f_{ij}^{*}s\left(p_{i},q_{j}\right)$$

Therefore, f* must yield a maximum objective value. For each feature *p*_*i*_ in sample *P*, we only need to find the most similar feature *q*_*j*_ in Q. Upon removing the first constraint, the second case is almost same, except the nearest neighbor search is reversed. If we combine the two relaxed solutions, denoted as *s*_1_(*P*, *Q*) and *s*_2_(*Q*, *P*), we can get an even tighter bound by taking the minimum of the two, *s*(*P*, *Q*) = *min*(*s*_1_(*P*, *Q*), *s*_2_(*Q*, *P*)). The time complexity of the relaxed optimization is *O*(*mn*), and it can be further reduced to O(*m*log*n*) by utilizing existing fast nearest neighbor retrieval [4], where *m* and *n* are the number of features in sample *P* and *Q*, respectively.

### E. APPLICATION FOR DISCOVERING SUBTYPES IN CANCER

Since somatic mutation data are extremely sparse in an entire high-dimensional gene group, it is very common that two clinically identical patients do not share any common mutation. So, the similarity between patients cannot be directly measured based on mutated genes using traditional distance metrics (e.g., Euclidean distance, Cosine Similarity). Therefore, for stratification of cancer into informative subtypes, existing methods based on traditional distance metrics cannot cluster patients with mutations very well. Much valuable information is available in public databases of human protein-protein, functional and pathway interactions, which are proved very useful to map the molecular pathways of cancer [41], [42]. Therefore, we can use our Feature Alignment Similarity to compute the similarity between patients. In this way, even if two patients do not have any mutations in common, they are likely to belong to the same cluster if their mutations reside in close network regions.

**FIGURE 3.**
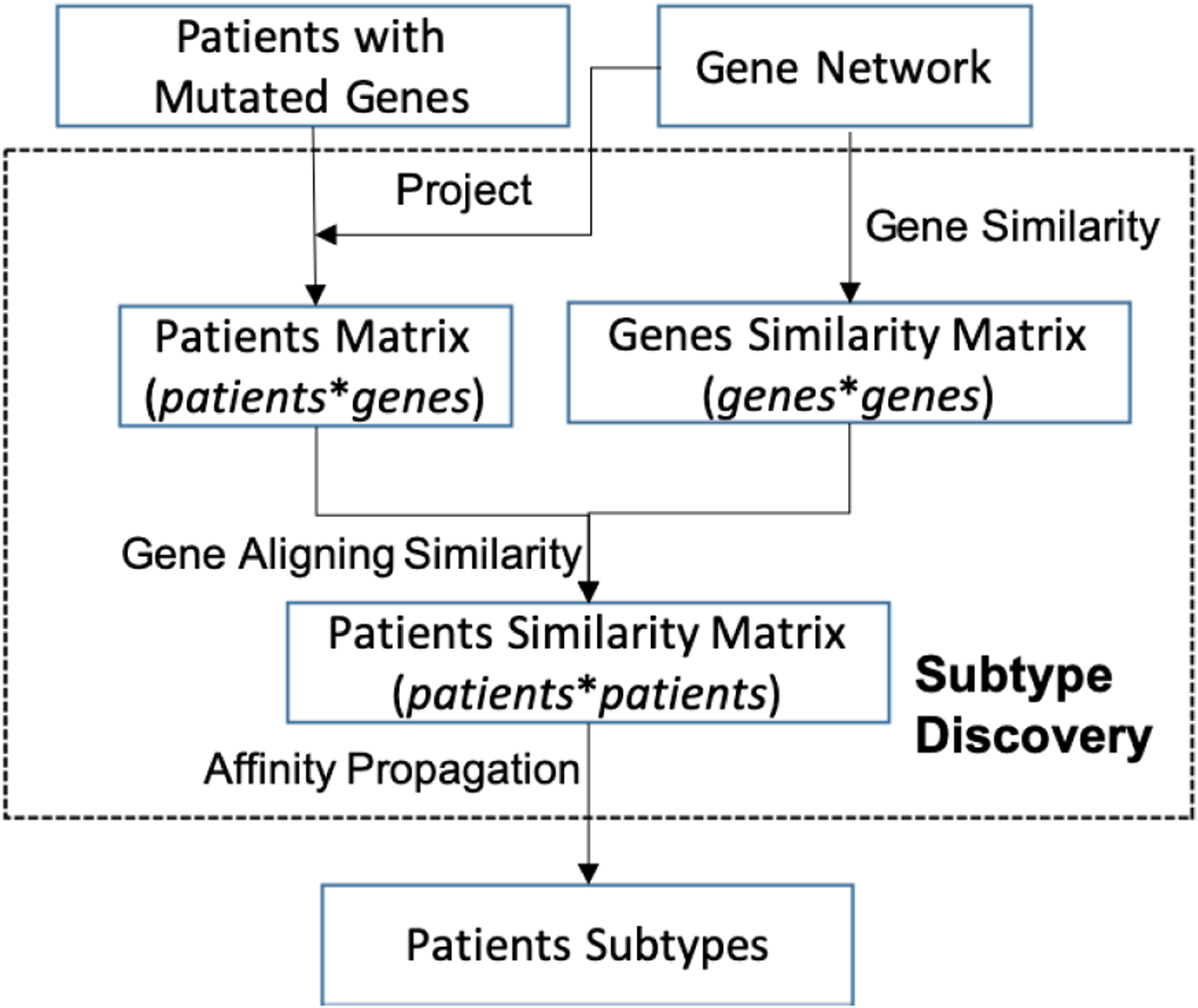
Framework of Network-based Affinity Propagation.

Figure 3 gives the flowchart of the approach to discover subtypes. Network-based Similarity combines somatic mutation data with a gene interaction network to produce a robust subdivision of patients into subtypes. First, we choose a subset of the genes by projecting each patient onto a gene interaction network from public databases [34]–[36]. Then, we need to compute the similarity between genes by using a gene network that finds the path of greatest similarity of two genes. After that, we need to match the vertices of two patients and apply our FAS metric to compute the similarity between patients. Finally, the similarity matrix of patients is used for cancer subtype discovery via Affinity Propagation [43]. Affinity Propagation is a clustering algorithm that takes measures of similarity between pairs of data points as input and simultaneously considers all data points as potential examples. The whole method referred to as Network-based Affinity Propagation (NetAP).

The above optimization is a special case of the Earth Mover’s Distance [44], [45], a well-known transportation problem for which specialized solvers have been developed [37], [46].

## IV. EXPERIMENT

The task of clustering cancer patients using tumor mutation information is difficult. A real-world cancer dataset typically has hundreds of samples, but the number of gene mutations can be well above 15,000 as shown in Table 1. That being said, cancer is a complex disease. Two cancer patients of the same cancer subtype may not share any common mutated genes. Therefore, many clustering methods cannot achieve good results if they calculate two samples’ similarity directly. Cancer has highly heterogeneous causes, and it is difficult to find a clear group of genes to determine subtypes. To better evaluation, we evaluate our NetAP algorithm using synthetic data and real-world data with different focuses:

1. **Evaluation using Synthetic Data**. How accurately does NetAP detect cancer subtypes with respect to various gene network structures? Does NetAP outperform the stateof-the-art algorithms used for cancer subtype discovery?
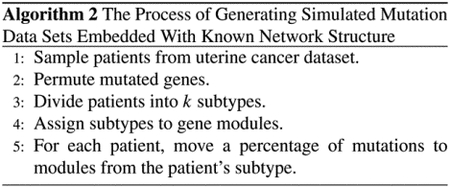
2. **Performance using Real-World Data**. Can NetAP detect cancer subtypes that are clinically meaningful? What is the impact of different gene networks on performance? Can NetAP identify cancer subtypes that cannot be detected by other clustering algorithms?

In our empirical study, we observe that AP is the strongest baseline clustering algorithm even though it does not use a gene network. A possible explanation is that the power of belief-propagation can better tune the center of each cluster. Hence, in our experiments, we chose AP to integrate with gene interaction networks for optimal performance.

We implemented NetAP in Matlab.^1^ All experiments were conducted on a Windows machine with an Intel 437 2.9 GHz CPU and 8GB memory. Table 2 and Table 3 show the details of the real-world uterine and lung cancer datasets as well as three gene interaction networks we used in our experiments.

### A. DATASET INFORMATION AND EXPERIMENT SETUP

#### Synthetic Data:

To test the accuracy of our method, we adopt a synthetic dataset [13] to mirror the biological characteristics of cancer and investigate the effectiveness of incorporating gene interaction networks. The process of generating a simulated mutation dataset is shown in Algorithm 2. The synthetic dataset adopts the structure of TCGA uterine tumor mutation data^2^ and the PathwayCommons gene interaction network [34]. First, mutation profiles are permuted, and patients are randomly divided into a predefined number of subtypes (*k* = 4). Then, we transferred a fraction of the mutations in each patient to fall within genes of a single ‘network module’ characteristic of that patient’s subtype, and the rest of mutations are left to occur randomly. Here, we set the ‘driver’ mutation frequency *f* varied from 1% to 15%, and we selected the size of network modules randomly from the whole network modules with size ranges 10–250 (see the paper [13] for details and justification for the range of *k*, *f* and *s*). Through this synthetic data, we measured the ability of NetAP to recover correct subtype assignments in comparison to other state-of-the-art methods including three methods not based on network knowledge and one method based on network knowledge.

#### Real-World Data:

High-grade uterine endometrial carcinoma and lung adenocarcinoma somatic mutational data were collected from The Cancer Genome Atlas (TCGA) data portal. Only mutation data generated using the high-quality Illumina GAIIx platform were saved for the following analysis, and patients with less than 10 mutations were removed for a fair comparison [13]. Patient mutation profiles are constructed as binary vectors such that a bit is set to 1 if the gene corresponding to that position in the vector is mutated in that patient. We follow the same somatic mutational data processing procedure as [13], [24].

#### Evaluation Metrics:

The clustering results on real-world data are evaluated using histological types provided by the TCGA data. Five metrics are used to measure the clustering performance: Normalized Mutual Information (NMI), Rand Index (RI), Adjusted Rand Index (AR), Chi-Square test and P-Value. NMI, RI and AR are widely used to evaluate the performance of clustering algorithms in data mining and machine learning [47], [48]. Chi-square test (Chi-Square) and P-Value are mostly used in statistics and bioinformatics [49]. For NMI, RI, AR, and Chi-square, a larger score indicates better clustering performance. For P-Value, a small value represents good clustering quality.

- Normalized Mutual Information (NMI) is a clustering validation metric that effectively measures the amount of statistical information shared by the predicted cluster assignments and ground truth, independent of absolute cluster labels. Two patients are assigned to the same cluster if and only if they are similar; thus, clustering can be viewed as a series of pair-wise decisions.
- Rand Index (RI) measures the percentage of clustering decisions that are correct. Rand Index can be adjusted for the chance clustering of elements, which will result in one of its variants called Adjusted Rand Index (AR). AR has a value between 0 and 1, and RI can have negative values.
- Chi-Square is used to determine whether there is a significant difference between expected clusters and observed clusters.
- P-Value can determine how significant clustering results are by performing a hypothesis test commonly used in statistics.

#### Existing State-of-the-Art Methods for comparison:

We compared our NetAP algorithm^3^ with Nonnegative Matrix Factorization (NMF) [16], Latent Dirichlet Allocation (LDA) [17], Affinity Propagation (AP) [43], and Network-based stratification(NBS) [13]. For each model, we set *K* as the real number of clusters of each dataset.

#### NMF [16]:

an unsupervised learning technique originally employed to decompose high-dimensional data $\in {\mathbb{R}}^{m\times n}$ into two non-negative matrices $W\in {\mathbb{R}}^{m\times k}$ and $H\in {\mathbb{R}}^{k\times n}$ whose product is an approximation of *A*. Here, *W* vector of coefficients can be interpreted as the *k* topic membership weights for the corresponding document. We use the open-source MATLAB implementation^4^ for NMF based on Euclidean distance.

#### LDA [17]:

a directed graphical model that models a document as a mixture of topics and a topic as a mixture of words. When LDA is used for text clustering, we choose the maximum value from a mixture of topics as its cluster label for each document. LDA based on Gibbs sampling is chosen as comparison [18].^5^ *α* and *β* of LDA are set as 0.1 and 0.1.

#### AP [43]:

a clustering algorithm that takes as input measures of similarity between pairs of texts and simultaneously considers all data points as potential examples. For AP, we use the “apcluster” package in R.^6^ Based on empirical observation, the Pearson correlation coefficient is chosen as the distance metric. We set parameter *λ* = 0.9 for AP.

#### NBS [13]:

a clustering algorithm that incorporates the information from gene networks into network-based non-negative matrix factorization [32]. The source code of NBS is provided in Hofree *et al*. [13].

#### Other Baselines:

more clustering algorithms are used for subtype discovery. Here, we choose these algorithms (SparseNMF [30], Kmeans [48], PAM [50] and Hierarchical [51]).

#### NetAP:

the proposed method by this paper. A gene network is a nearest neighbor network derived from the graph Laplacian of an influence distance matrix [42] that comes from the original gene interaction network, *G* = {*V*, *E*}. To obtain the gene network in NetAP, we experimented with neighbor counts ranging from 5 to 50 to include in the nearest network, and we observed only small changes in outcome. For the work shown in this paper, 11 most influential neighbors of each gene in the network as determined by network influence distance were used.

#### Gene Interaction Network:

To evaluate the impact of different gene interaction networks, three major gene interaction databases are used: PathwayCommons [34], STRING [35] and HumanNet [36]. PathwayCommons^7^ includes gene interaction information extracted from multiple gene interaction databases, and its focus is on physical protein-protein interactions. We excluded all non-human genes and interactions from the PathwayCommons network in our experiments. STRING^8^ collects protein-protein interactions from expression data analysis and medical literature using text mining methods. HumanNet^9^ is built by a modified Bayesian integration from multiple organisms. Only the top 10% interactions of STRING and HumanNet are used in our experiments to reduce noise. Table 2 summarizes the number of genes and interactions, and the numbers in parentheses are what we used in our experiments.

### B. EVALUATION ON SYNTHETIC DATA

**FIGURE 4.**
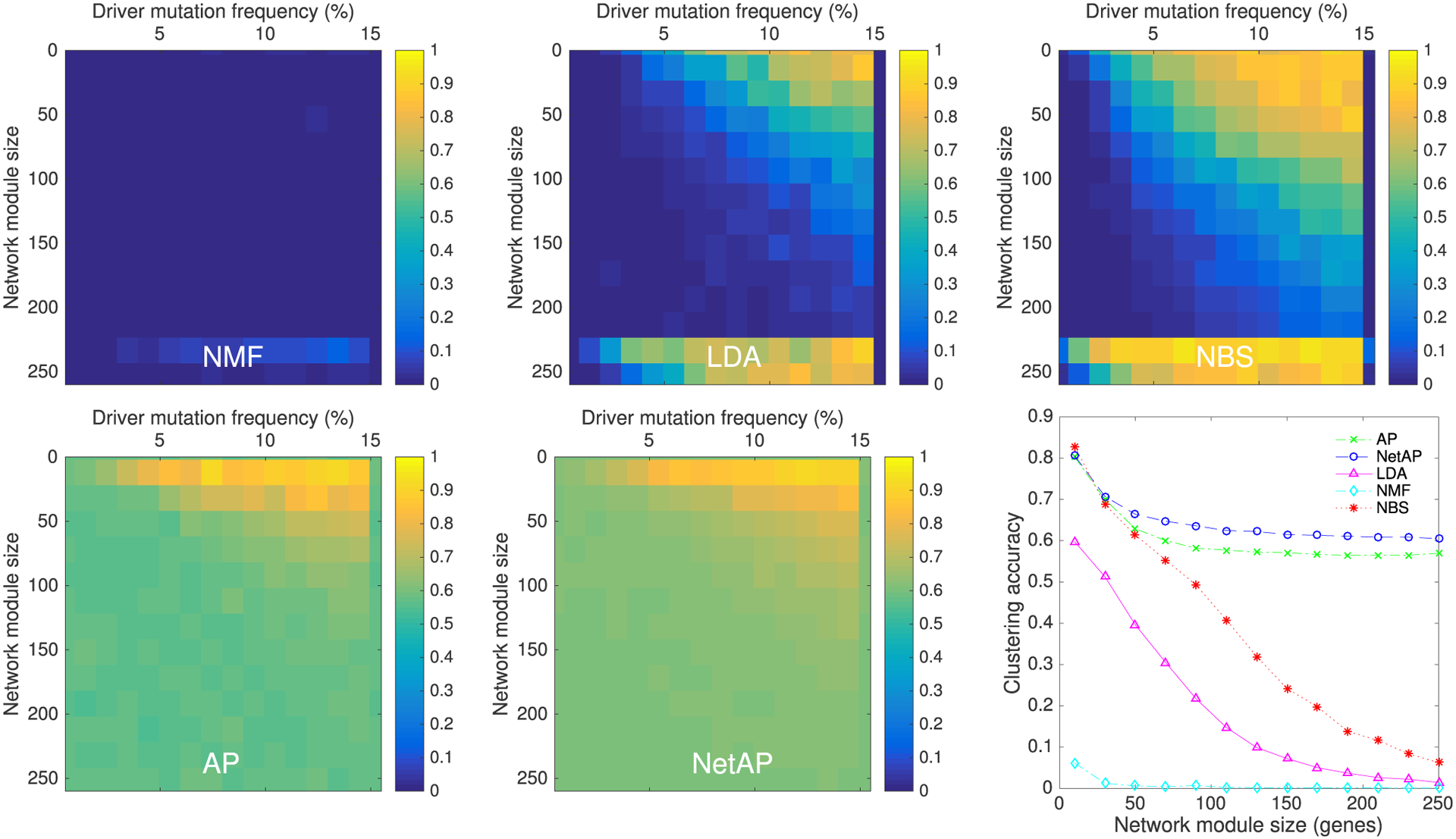
Exploring performance of NetAP on synthetic data through varying driver mutation frequency and network module size (the first five sub-figures). We sum all accuracies of different driver mutation frequency (0.01 to 0.15) by varying network module size (the last sub-figure).

In Figure 4, the comparison is done among NMF, AP, LDA and NBS on synthetic data using AR metric. We run each algorithm 20 times, and all results of these methods are the average value of these 20 runs per experimental setting. First, we investigate how NetAP performance is affected by driver mutation frequency and network module size. The first 5 subfigures of Figure 4 show the results of NMF, LDA, NBS, AP and NetAP. LDA and NBS are sufficient for stratification at high mutation frequencies and small module size, in which there is high overlapping in mutations among patients of the same subtype. For large network modules and small driver mutation frequency, LDA and NBS cannot accurately recover the correct subtypes. However, AP and NetAP were able to accurately recover correct subtypes for a much larger range of both variables. Compared with AP, the experimental results demonstrate the effectiveness of NetAP.

For a better demonstration of the results, we sum all accuracies of different driver mutation frequency under each network module size, which is shown in the last sub-figure of Figure 3 (bottom right). The results demonstrate that NetAP can effectively detect cancer subtypes with respect to various driver mutation frequencies and network module sizes, especially for large network modules, as these can be associated with any of numerous different mutations across the patient population. As module size decreases, the chance of observing same mutated gene in patients of the same subtype increased, and some existing cluster algorithms performed better (LDA and NBS). AP that does not utilize gene interaction networks has the closest performance to NetAP.

### C. EFFECTIVENESS ON REAL-WORLD DATA

**FIGURE 5.**
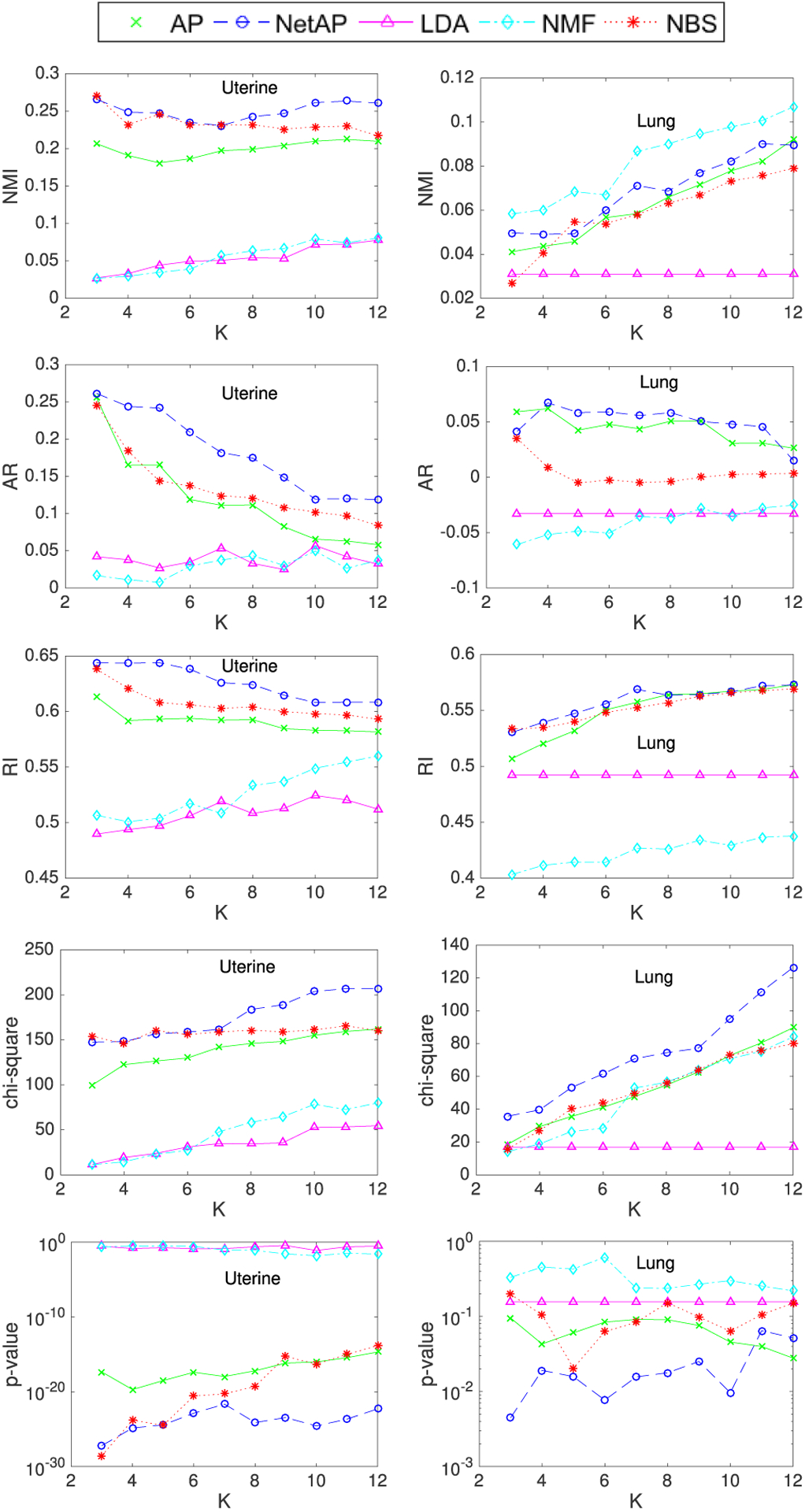
Performance of NetAP compared to NMF, LDA, AP, and NBS with different values of *K* using NMI, Rand index, Adjusted Rand Index, Chi-square and P-value metrics on uterine and lung Cancer. NetAP is our proposed method. For P-Value, the smaller the better. For others, the larger the better.

In Figure 5, we demonstrate that NetAP can detect more statistically significant cancer subtypes in uterine cancer and lung cancer datasets. NBS and NetAP use the PathwayCommons network.For the othertwo networks (STRING and HumanNet), we will discuss their impact on NBS and NetAP in next section. The results of all methods are illustrated assuming 10 different numbers of subtypes from 3 to 12. From the results on uterine cancer, NetAP and NBS perform better than AP, NMF and LDA, which confirms that gene network knowledge helps improve the clustering performance for uterine cancer. We observe that NetAP consistently outperforms NBS. Notably, NetAP achieves almost 30% improvement on AR metric over NBS.

On lung cancer, NetAP performs better than other methods in terms of AR, RI, Chi-square and P-value metrics, except NMI metric. Similar to the results on uterine cancer, NetAP performs most similarly to NBS. However, although NBS still outperforms NMF, it has similar performance with AP that does not take advantage of gene network structure. We suspect that NMF-based methods (NBS is based on NMF) cannot deal with extremely sparse data such as somatic mutation data that has lots of 0s and few 1s, even though incorporating network information can help to alleviate the sparseness problem to a certain degree.

Table 3 shows that the well-established clustering algorithm SparseNMF (NMF using L1 regularization), Kmeans, PAM, Hierarchical clustering algorithms have almost identical or worse performance than random assignment. In our work, we assume that cancer patients belonging to one subtype are more likely to share a similar network subregion. Network-based NBS (also NMF based) achieves better results than NMF, and NetAP outperforms all other methods that we compared against in real-world data. NMF and its variations do not work well due to sparse and heterogeneous characteristics of somatic mutation feature space.

In summary, we can conclude that NetAP is the most appropriate clustering algorithm for clustering gene mutations, which can produce a robust division of patients into subtypes from somatic mutation profiles combining gene interaction network. Because NBS and NetAP are the only two algorithms using gene network, we will compare them in more detail that use two more gene networks.

#### 1) PERFORMANCE OF NetAP COMPARED TO MORE BASELINES

We chose four existing methods (NMF, LDA, AP, NBS) in the previous experiment. To fully assess the effectiveness of our method, we conducted more experiments to compare with other clustering algorithms (SparseNMF [30], Kmeans [48], PAM [50] and Hierarchical [51]). Due to the space limit, we only show the results on uterine data. For conciseness, we only show the results using NMI metric in Table 3. “Random” refers to the result by random drawing. The performance of these existing methods is very similar to “Random”, which means all these methods are not effective for somatic mutation stratification due to the extreme sparseness of somatic mutations. Therefore, incorporating knowledge of a gene network to reduce sparseness is very important for identifying subtypes from somatic mutation data.

#### 2) IMPACT OF GENE NETWORKS

**FIGURE 6.**
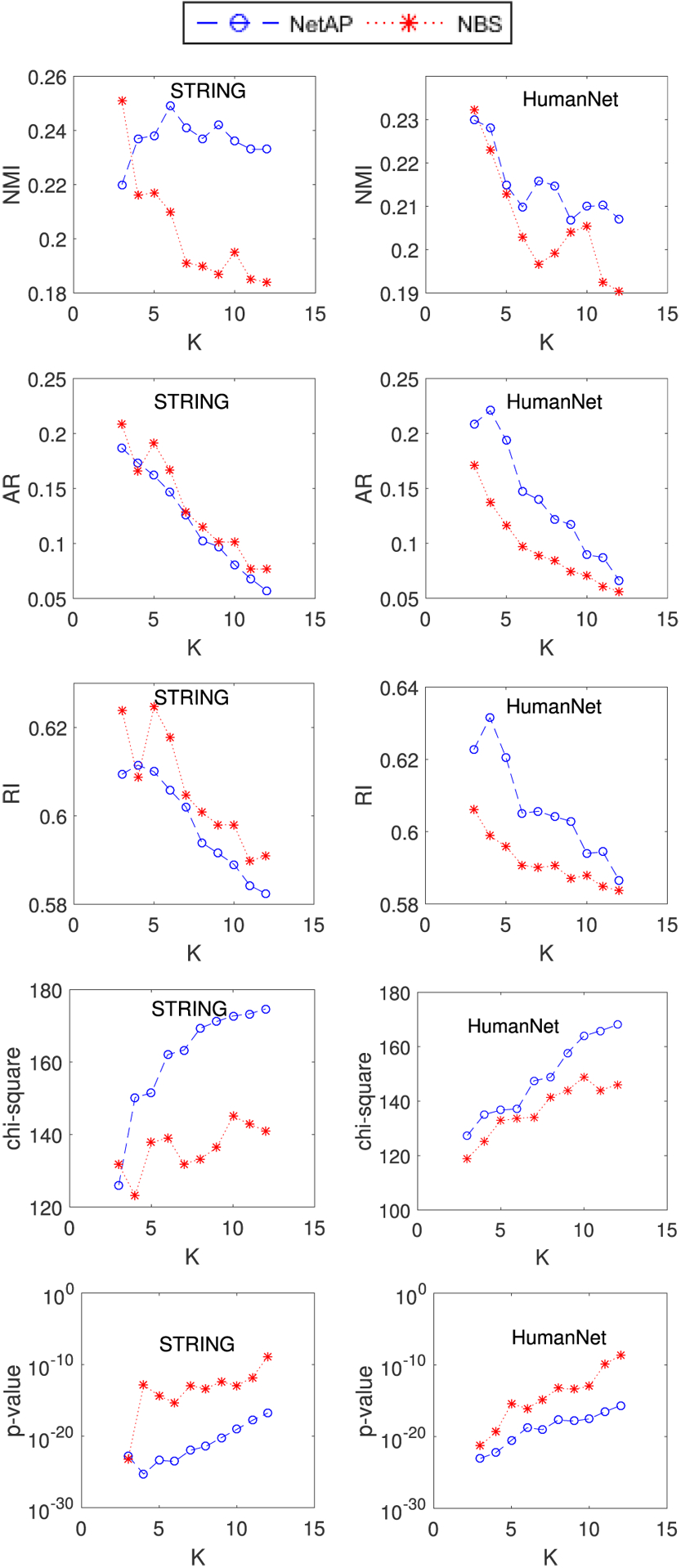
Performance of NBS and NetAP on the other two human networks (STRING and HumanNet) with respect to different values of *K*. For P-value, the smaller the better. For others, the larger the better.

Figure 6 shows the performance of NetAP and NBS on the uterine cancer dataset by incorporating the other two networks (STRING and HumanNet) with different numbers of subtypes (*K* = 3, 4, …, 12) using five metrics (NMI, RI, AR, Chi-square, P-value). Clearly, NetAP works better than NBS on these two gene interaction networks in general, except on AR and RI metrics using STRING network. Especially, when increasing the number of subtypes *K*, NetAP can achieve better results than NBS. Similar to the results that use the PathwayCommons network, the experimental results give further evidence that our method NetAP is more robust for subtype identification than NBS.

As NetAP is naturally dependent on gene interaction networks, we examine how different gene networks affect the quality of NetAP with NMI metric. We chose the following three gene networks: PathwayCommons, STRING and HumanNet. Figure 6 shows the results of NetAP with different gene networks on uterine cancer dataset. When varying subtypes from 3 to 12, NetAP using PathwayCommons or STRING performs better than NetAP using HumanNet. Additionally, NetAP using PathwayCommons outperforms NetAP using STRING. In conclusion, the performance of NetAP will vary when it incorporates different gene networks. This new finding indicates that PathwayCommons can provide strong genetic traits for usage on cancer subtype discovery.

#### 3) IDENTIFYING SUBTYPES

**FIGURE 7.**
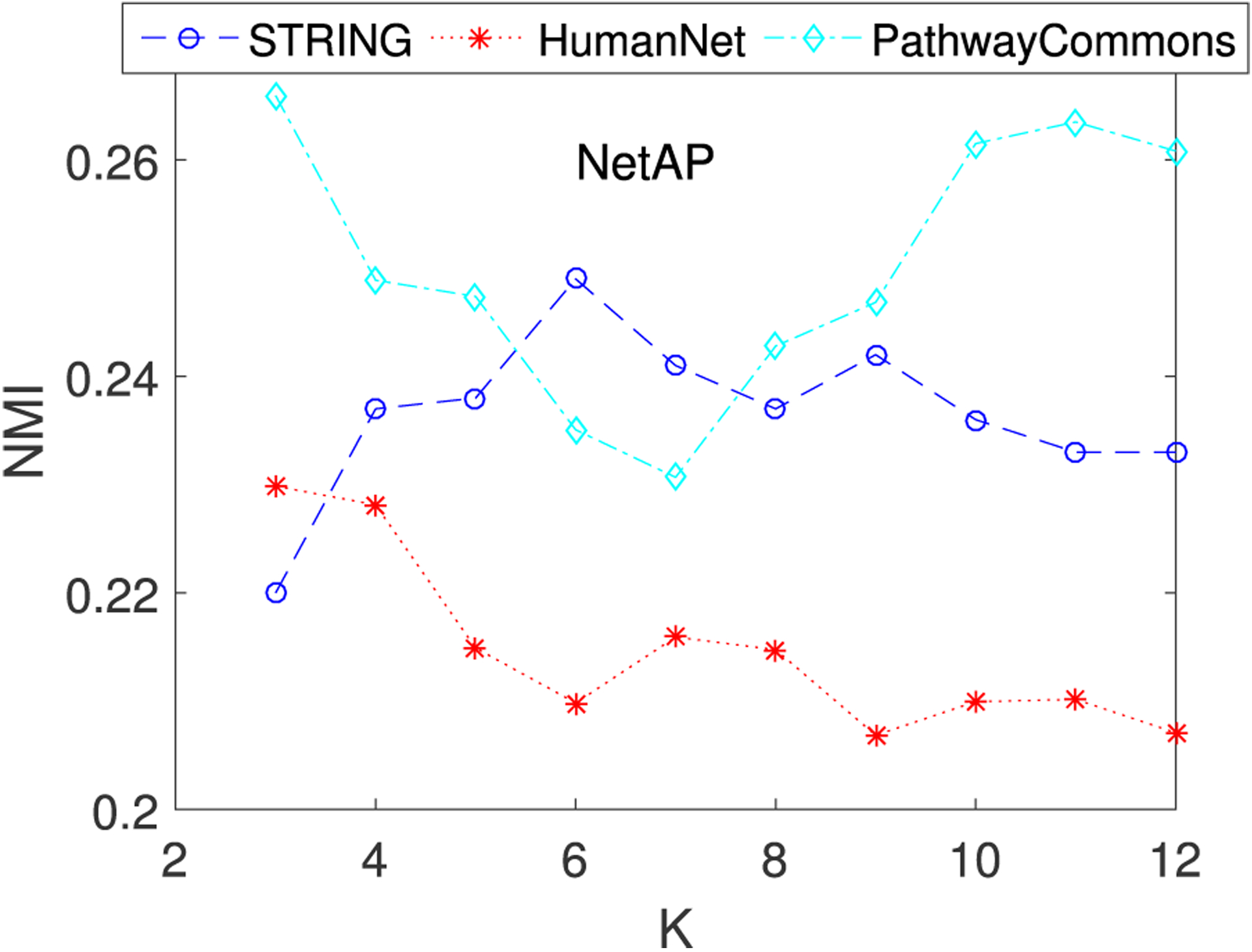
Performance of NetAP with three gene networks (PathwayCommons, STRING and HumanNet) using NMI on Uterine cancer.

To assess the biological significance of the identified subtypes, we examine whether they correlate with observed clinical data. Figure 7 shows the results of NMF, LDA, AP, NetAP and NBS with recorded subtypes on a histological basis. We can see that NetAP subtypes are more closely associated with recorded subtypes on a histological basis than other algorithms. NMF and LDA cannot separate “serous adenocarcinoma type” and “endometrioid type” from the data set. NBS can only extract one subtype “serous adenocarcinoma type”. NetAP and AP can separate two subtypes “serous adenocarcinoma type” and “endometrioid type”. Furthermore, NetAP has higher accuracy than AP.

## V. CONCLUSION

In this paper, our goal is to propose a novel similarity measure that computes the similarity between samples by incorporating the knowledge of interaction networks to overcome data sparseness problem. Our metric does not directly compute the similarity between two samples but measures the similarity by the similarity from the embedded features of one sample to another after the two samples are projected into feature interaction network. In this way, although two samples do not share one common feature, they are likely to belong to the same clustering when their mutations share the similar network regions. When applied to the discovery of cancer subtypes, our approach demonstrates effectiveness and efficiency on synthetic and uterine adenocarcinoma datasets along with three popular gene networks using five different metrics. In the future, we plan to integrate multiple layers of information beyond somatic mutations (e.g. CNVs, transcriptome, etc.) into our method for better subtype identification.

## Figures and Tables

**FIGURE 8.**
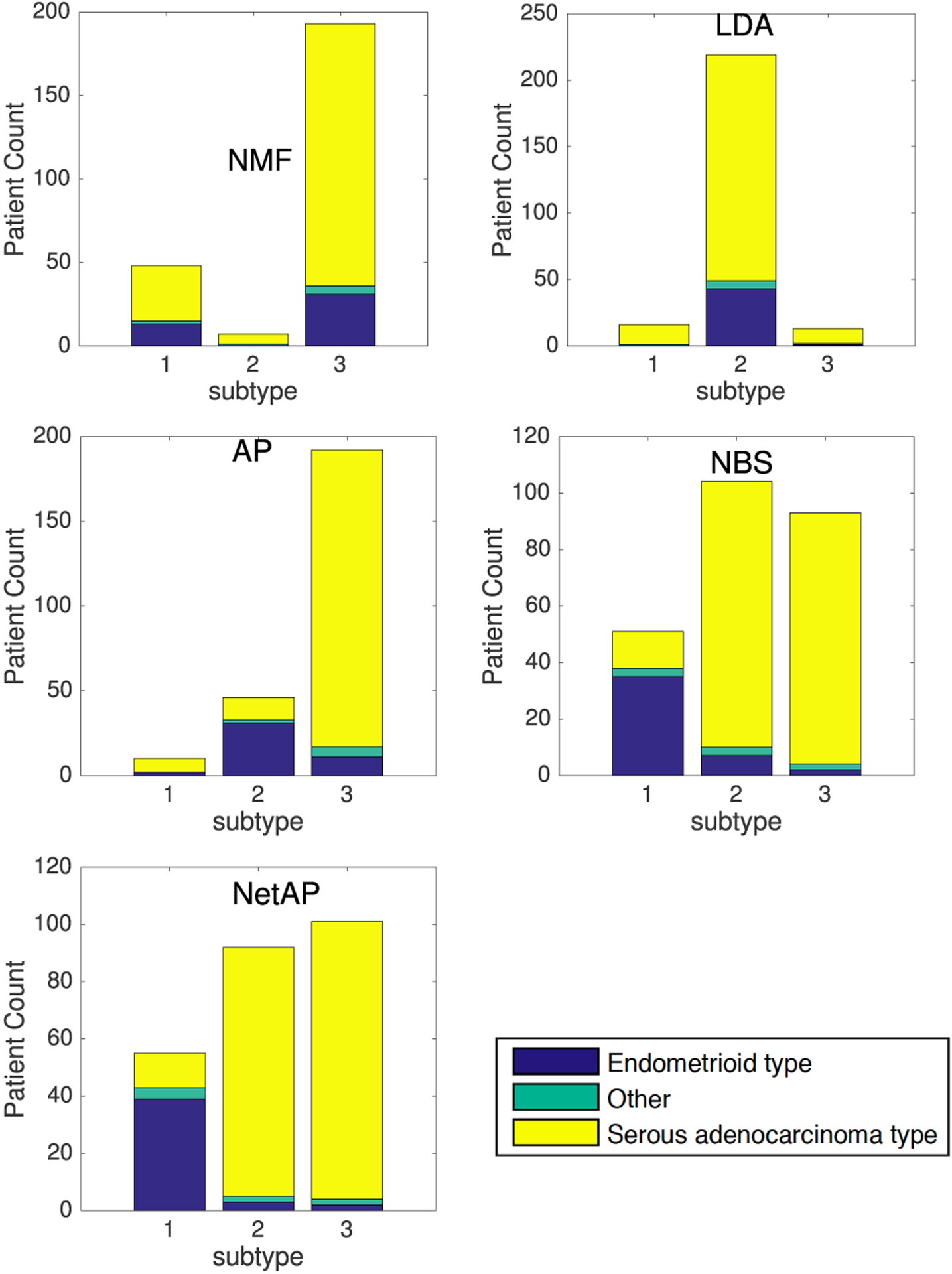
Summary of Histological types for each subtype on Uterine Cancer.

**TABLE 1.** Summary of Uterine and Lung cancers.

Dataset	#	SIZE	AVG
Uterine	248	17968	612.96
Lung	304	15967	326.83

#: the number of patients, SIZE: the number of genes, AVG: the average mutated genes of each patient

**TABLE 2.** Summary of gene interaction networks.

	Nodes	Edges
PathwayCommons	14,355 (2814)	507,757 (33,757)
STRING	16,569 (12,233)	1,638,830 (164,034)
HumanNet	16,243 (7,949)	476,399 (47,641)

**TABLE 3.** Performance of NetAP and other clustering methods on uterine cancer using NMI.

K	3	4	5
Random	0.0138	0.0199	0.0245
SparseNMF	0.0247	0.0141	0.0198
Kmeans	0.0332	0.1035	0.1040
PAM	0.0136	0.0708	0.1073
Hierarchical	0.0107	0.0708	0.1073
NetAP	0.2659	0.2488	0.2473

## Footnotes

1 The source code can be downloaded at https://github.com/qiang2100/NetAP

2
https://tcga-data.nci.nih.gov/tcga/

3 Our code is open-sourced at https://github.com/qiang2100/NetAP.

4
https://sites.google.com/site/nmftool/

5
http://psiexp.ss.uci.edu/research/programs_data/toolbox.htm

6
https://cran.r-project.org/web/packages/apcluster/index.html

7
www.pathwaycommons.org/pc/

8
www.string-db.org/

9
www.functionalnet.org/humannet/
